# Prognostic factors of Guillain-Barré syndrome: a 111-case retrospective review

**DOI:** 10.1186/s41016-018-0122-y

**Published:** 2018-06-18

**Authors:** Yitao Zhang, Yanyin Zhao, Yi Wang

**Affiliations:** Department of neurology, Huashan Hospital affiliated to Fudan University, 12 M.Wulumuqi Rd, Jina’an District Shanghai, People’s Republic of China

**Keywords:** Guillain-Barre syndrome, outcome, Prognostic factors

## Abstract

**Background:**

To identify the predictive factors associated with worse prognosis in the Guillain-Barré syndrome (GBS), which can be helpful to fully evaluate the disease progression and provide proper treatments.

**Methods:**

Clinical data of 111 GBS patients who were diagnosed from 2010 to 2015 were collected and retrospectively analyzed.

**Results:**

Patients with diabetes (*P*=0.031), high blood pressure at admission (*P*=0.034), uroschesis (*P*=0.028), fever (*P*<0.001), ventilator support (*P*<0.001) during hospitalization, disorder of consciousness (*p*=0.007) and absence of preceding respiratory infection(*P*=0.016) were associated with worse outcome at discharge, while abnormal sensation, ataxia, weakness and decrease of tendon reflex seemed not correlated with the Medical Research Council(MRC) score at discharge. Compared with the subtype of acute inflammatory demyelinating polyneuropathy, prognosis of Miller-Fisher syndrome (*p*<0.001) and cranial nerve variant (*p*<0.038) were better, but prognosis of acute motor axonal neuropathy(AMAN) was worse (*p*<0.032). Laboratory examinations at admission showed that hyperglycemia (*P*=0.002), high leukocyte count (*P*=0.010), hyperfibrinogenemia (*P*=0.001), hyponatremia (*P*=0.020), hypoalbuminemia (*P*=0.005), abnormal hepatic (*P*=0.048) and renal (*P*=0.009) functions were associated with poorer prognosis at discharge, while albuminocytologic dissociation in cerebrospinal fluid, GM1 and GQ1b antibody showed no correlation with the MRC score at discharge. γ-Globulin and glucocorticoid therapies showed no difference in the MRC score at the discharge.

**Conclusions:**

AMAN, diabetes, high blood pressure, uroschesis, high body temperature, ventilator support, consciousness disorder, absence of upper respiratory tract preceding infection, hyperglycemia, hyponatremia, hypoalbuminemia, high leukocyte count, hyperfibrinogenemia, abnormal hepatic and renal function were demonstrated as poor prognostic factors.

## Background

Guillain-Barre syndrome (GBS) is a set of clinical syndromes with a common pathophysiological basis, and is usually considered to be an immune-mediated disorder of the peripheral nervous system [1, 2]. GBS is usually characterized by symmetrical flaccid paralysis with areflexia, which usually reaches a maximum severity within four weeks [3, 4]. However, recent studies suggest that some patients with GBS had normal or hyperreflexia [5, 6], and a wide range of motor, sensory and autonomic symptoms could also be found from GBS patients [7, 8]. The reported mortality of GBS in whole population ranged from 0.89 to 1.89 cases (median 1.11) per 100,000 people [9], which makes GBS the most common cause of acute flaccid paralysis currently. Intravenous immunoglobulin (IVIG) and plasma exchange (PE) were proven to be effective therapeutic method for GBS [10, 11] and are now widely used clinically which lower the mortality rate effectively, making most of the patients a complete functional recovery or with minimal deficits [12]. However, there are still some cases with bad prognosis and sequelae such as decreased mobility, severe long-term fatigue syndrome and chronic pain [12]. The reported mortality in GBS patients now varies between 3% and 7% [13–15]. With this study, we aimed to identify the predictive factors associated with worse prognosis in the GBS.

## Methods

We retrospectively reviewed 111 patients diagnosed GBS who were treated in the neurology department of Huashan Hospital and Huashan North Hospital affiliated to Fudan University from 2010 to 2015. The patients were diagnosed based on clinical features and electrophysiological findings. Demographic, clinical information of all patients was recorded and reviewed.

The Hughes Functional Grading Scale (HFGS) score was used to assess functional disability, which was defined

as follows: 0, healthy state; 1, minor symptoms and capable of running; 2, able to walk 5 m or more without assistance but unable to run; 3, able to walk 5 m across an open space with help; 4, bedridden or chairbound; 5, requiring assisted ventilation for at least part of the day; 6, dead.

Medical Research Council (MRC) sum score, valuing the strength from 0 to 5 in 4 muscles (proximal and distal) in both upper and lower limbs on both sides, so that the score ranged from 40 (normal) to 0 (quadriplegic).

At admission, the patients’ blood samples were drawn and lumbar puncture was performed to collect the cerebrospinal fluid. Laboratory exam data were collected and analyzed.

Some patients also received the electromyogram examination during hospitalization. According to the electromyogram examination, patients with demyelination and axonal damage were classified.

All the demographic, clinical, laboratory exam and electromyogram exam data were all collected from the department of medical record of Huashan Hospital and Huashan North Hospital affiliated to Fudan University.

The study was approved by the Ethics Committee of Huashan Hospital affiliated to Fudan University.

MRC sum score was regarded as the estimation of prognosis, as previous reports revealed that the MRC sum score has predictive value for prognosis which is more accurate than the GBS disability score [16, 17]. We assume that the higher MRC score at discharge means better prognosis.

Statistical analysis was performed by using SPSS software (version 24.0). Classification variables analysis was performed with the use of Kruskal-Wallis and U Mann-Withney with the non-parametric ones. Univariate analysis was used to identify the factors associated with poor prognosis, which were further analyzed by Logistic regression analysis for predictors independently related to the poor prognosis. A *p* value less than 0.05 was considered statistically significant. When doing the Logistic regression analysis, patients who scored 33 or more in the MRC score at discharge were classified as patients with good prognosis, and the rest were classified into the poor prognosis group.

## Results

One hundred eleven cases of GBS patients met the inclusion criteria of the study, including 65 men (mean age: 41.88±17.38 years, ranged from 14 to 82 years) and 46 women (mean age:49.28±14.13 years, ranged from 21 to 80 years) . 67 patients (60.36%) were diagnosed as acute inflammatory demyelinating polyneuropathy (AIDP), 9 patients (8.11%) were diagnosed as acute motor axonal neuropathy (AMAN), 24 patients (21.62%) were diagnosed as Miller-Fisher syndrome (MFS), 8 patients (7.20%) were diagnosed as cranial nerve variant (CNV), 1 patient (0.90%) was diagnosed as Bickerstaff's brainstem encephalitis overlaps with Guillain-Barre syndrome (BBE-GBS) and 2 patients’ classifications were not clear.

5 patients were diagnosed as type 2 diabetes mellitus before. 61.8% patients had a preceding infection before the onset of GBS, which include 39 cases of upper respiratory infection, 9 case of diarrhea, 3 cases of both upper respiratory infection and diarrhea, 4 cases of virus infection, 13 cases of other infection such as postoperative infection.

A motor disorder at the admission was the most common symptom, according to HFGS score, 63.9% retained the ability to walk (grades 1, 2 and 3), while the remaining 36.1% showed a severe disability (grades 4, 5 and 6) because of motor disorder and non-motor symptoms such as respiratory difficulty. 50.5% cases showed cranial nerve involvement, including glossopharyngeus nerve and vagus nerve(21, 18.91%), facials nerve (17, 15.32%), oculomotor nerve(24, 21.62%), and abducens nerve(26, 23.42%). Non-motor symptoms were also described frequently and the most frequent one was sensory paralyses (52.18% of all the patients), followed by neuropathic pain (19.8%), ataxia (21.05%), retention of urine (8.1%), and dry skin (3.6%). 15.3% of patients received mechanical ventilation in hospital. According to the MRC score at discharge, 83 patients (74.8%) got more than 30 points, 21 (18.9%) ranged from 10 to 30, 4(3.6%) got lower than 10 points, and 3 (2.7%) patients died.

Several clincial features-related prognosis predictors of patients with GBS (as shown in Table 1) were found. Prognosis of GBS patients with diabetes was worse compared with those without diabetes (*p*=0.031). GBS patients with high blood pressure at admission have worse outcome at discharge (*p*=0.034). Different subtypes of GBS have different prognosis. Compared with the AIDP subtype which is the most common subtype in our study, the prognosis of MFS (*p*<0.001) and CNV (*p*<0.038) were better, and the prognosis of AMAN was worse (*p*<0.032). Interestingly, we also discovered that the preceding upper respiratory infection is related to better prognosis (*p*=0.016). The MRC scores of patients who had preceding upper respiratory tract infection were 35.82±8.0 at discharge, while GBS patients with no preceding infection scored 30.80±11.96.

**Table 1:** Clinical features related prognosis predictors of patients with GBS

	Number of patients (%)	MRC score at discharge	Comparison^*^ (*P*-value)
Type II Diabetes			0.031*
With	5 (4.50%)	22.4±11.8	
Without	96 (95.50%)	33.83±9.85	
High blood pressure			0.034*
With	13 (11.71%)	24.08±16.73	
Without	98 (88.29)	34.55±8.33	
GBS subtype			<0.001*
AIDP	67 (60.36%)	31.45±10.877	
AMAN	9 (8.11%)	23.89±12.129	
MFS	24 (21.62%)	39.88±0.448	
CNV	8 (7.21%)	38.00±3.546	
BBE-GBS	1 (0.90%)	40.00±0.000	
GBS subtype			0.032*
AIDP	67 (60.36%)	31.45±10.877	
AMAN	9 (8.11%)	23.89±12.129	
GBS subtype			<0.001*
AIDP	67 (60.36%)	31.45±10.877	
MFS	24 (21.62%)	39.88±0.448	
GBS subtype			0.038*
AIDP	67 (60.36%)	31.45±10.877	
CNV	8 (7.21%)	38.00±3.546	
GBS subtype			0.209
AIDP	67 (60.36%)	31.45±10.877	
BBE-GBS	1 (0.90%)	40.00±0.000	
Preceding infection			0.042*
Upper respiratory tract infection	39 (35.14%)	35.82±8.003	
Diarrhea	9 (8.11%)	26.56±2.330	
Upper respiratory tract infection and diarrhea	3 (2.70%)	37.00±3.606	
Other virus	4 (3.60%)	37.25±4.856	
Other	13 (11.72%)	31.40±9.044	
Without	43 (38.74%)	30.80±11.96	
Preceding infection			0.0016*
Upper respiratory tract infection	39 (35.14%)	35.82±8.003	
without	43 (38.74%)	30.80±11.96	
Preceding infection			0.217
Diarrhea	9 (8.11%)	26.56±2.330	
Without	43 (38.74%)	30.80±11.96	
Preceding infection			0.552
Upper respiratory tract infection and diarrhea	3 (2.70%)	37.00±3.606	
Without	43 (38.74%)	30.80±11.96	
Preceding infection			0.014*
Upper respiratory tract infection	39 (35.14%)	35.82±8.003	
Diarrhea	9 (8.11%)	26.56±2.330	

*p values<0.05 are considered significant.

We also found some symptoms and signs during hospitalization-related prognosis predictors of patient with GBS (as shown in Table 2). Prognosis of GBS patients with urine retention was worse compared with that of normal urinating function (*p*=0.028). Prognosis of GBS patients with high body temperature (*p*<0.001), of those with conscious disorder was worse (*p*=0.007), of who needed mechanical ventilation (*p*<0.001) and stayed in intensive care unit (ICU) (*p*<0.001) were worse. What’s more, the higher of the MRC score at admission was, the better of the prognosis at discharge would be (p<0.001) according to the MRC score at discharge.

**Table 2:** Syndromes and signs during hospitalization related prognosis predictors of GBS patients

	Number of patients(%)	MRC score at discharge	Comparison^*^ (*P*-value)
Retention of urine			0.028*
With	9 (8.11%)	26.88±12.955	
Without	102 (91.89%)	33.81±9.828	
Body temperature			<0.001*
Normal	86 (77.48%)	35.74±6.674	
Abnormal	24 (21.62%)	24.35±15.275	
Use of mechanical ventilation			<0.001*
Yes	17 (15.32%)	17.75±12.434	
No	93 (83.78%)	35.91±6.931	
Consciousness			0.007
Normal	101 (90.99%)	16.25±18.468	
Disorder	9 (8.11%)	34.75±7.921	
ICU			<0.001*
Need	36 (32.43%)	26.91±13.744	
Do not need	65 (58.56%)	36.68±10.311	
The MRC score at admission			<0.001*
More than 30	65 (58.56%)	38.80±2.601	
10 to 30	41 (36.94%)	27.27±10.703	
Less than 10	5 (4.50%)	12.60±13.334	

*p values<0.05 are considered significant.

We also tried to find whether different treatments lead to different prognosis (as shown in Table 3). Among all the 111 patients, 40 patients (36.0%) received IVIg only, 11 patients (9.9%) received corticosteroids therapy only, 58 patients (52.3%) received both IVIg and corticosteroids therapy, 1 (0.9%) received all the IVIg, corticosteroids, and plasms exchange therapies, and 1 (0.9%) refused all these treatments. According to the MRC score at discharge, the prognostic difference among different therapeutic modalities were not significant.

**Table 3:** Treatment-related prognosis predictors of patients with GBS

	Number of patients(%)	MRC score at discharge	Comparison^*^ (*P*-value)
Treatment			0.975
IVIg	40 (36.04%)	32.41±14.330	
IVIg plus glucocorticoid	57 (51.25%)	33.63±8.557	
Treatment			0.175
IVIg	40 (36.04%)	32.41±14.330	
Glucocorticoid	11 (9.91%)	37.55±3.616	
Duration between onset and treatment			0.181
Less than 7 days	55 (49.55%)	32.22±10.906	
7 to 14 days	30 (27.03%)	33.43±10.897	
More than 14 days	23 (20.72%)	35.61±7.316	

*p values<0.05 are considered significant.

Some laboratory examination-related early predictors of a low MRC score at discharge, which was associated with a poor prognosis, were found (as shown in Table 4). All the laboratory examinations whose results analyzed were conducted at admission. Prognosis of GBS patients with hyperglycemia(*p*=0.002), hyponatremia that serum sodium level lower than 135mmol/L (*p*=0.020), and serum albumin level lower than 40g/L(p=0.005), were worse. Further logistic regression analyses revealed that higher the white blood cell count was, the lower the MRC score would be, indicating that higher white blood cell count was a possible predicator of poor prognosis. The same result occurred when plasma fibrinogen was taken into logistic regression analyses, that the higher the plasma fibrinogen was, the lower the MRC score would be. Prognosis of GBS patients with abnormal hepatic or renal function were worse. The differences were significant (*p*=0.048 and *p*=0.009 respectively). Patients with normal level of alanine aminotransferase (ALT) and aspartate aminotransferase (AST) scored 34.73±8.72, and those with abnormal level of ALT or AST scored 29.52±12.63. Patients with normal level of blood urea nitrogen (BUN) and Serum creatinine (Scr) scored 34.39±9.66, and those with abnormal level of BUN or Scr scored 28.86±11.52.

**Table 4:** Laboratory Examination-related prognosis predictors of patients with GBS

	Number of patients(%)	MRC score at discharge	Comparison^*^ (*P*-value)
Serum sodium concentration			0.020*
Low	24 (21.62%)	28.57±12.73	
Normal	85 (76.58%)	34.93±8.34	
Fasting glucose level			0.028*
High	27 (24.32%)	27.15±14.136	
Normal	78 (70.27%)	35.74±6.689	
Serum albumin level			0.005*
Normal	55 (49.55%)	36.69±5.231	
Low	54 (48.65%)	30.34±12.075	
White blood cell count			0.010*
Low	8 (7.21%)	36.00±7.303	
Normal	76 (68.47%)	35.03±7.806	
High	23 (20.72%)	26.25±14.435	
Hepatic function			0.048*
Elevated ALT/AST level	32 (28.83%)	29.52±12.630	
Normal ALT&AST level	78(70.27%)	34.73±8.719	
Renal function			0.009*
Elevated BUN/Scr level Normal BUN&Scr level	22 (19.82%) 88 (79.28%)	28.86±11.521 34.39±9.657	
Plasma fibrinogen			0.001*
High	23 (20.72%)	26.35±13.533	
Normal	59 (53.15%)	35.16±8.875	

*p values<0.05 are considered significant.

The significant association between albuminocytologic dissociation in cerebrospinal fluid (CSF) with the poor prognosis was not found from our study. Neither did the CSF & serum level of GM1 or GQ1B antibody. No significant MRC score at discharge differences between demyelination and axonal damage which were classified by electrophysiological findings.

Factors associated with poor were further analyzed by Logistic regression analysis. The result showed that low MRC score when admission, abnormal renal function, abnormal blood glucose level, and the subtype of AMAN are independently related to the poor prognosis with statistical differences (as shown in Table 5).

**Table 5:** Logistic regression analysis to find predictors independently related to the poor prognosis

Factors	Regression coefficient	Wals	Comparison^*^ (*P*-value)	Odds ratio
Type II Diabetes	-2.138	0.037	0.848	0.118
AMAN	-2.299	7.605	0.006*	0.100
AIDP	-0.895	3.014	0.083	0.408
Preceding upper respiratory tract infection	0.844	2.642	0.104	2.326
Retention of urine	-0.556	0.102	0.750	0.573
Abnormal body temperature	-3.791	1.754	0.185	0.023
Use of mechanical ventilation	-0.609	0.065	0.798	0.544
Consciousness	1.510	0.346	0.556	4.526
Need ICU	-3.894	1.578	0.209	0.020
MRC score at admission	2.362	4.668	0.031*	10.609
Without abnormal serum sodium concentration	0.503	0.912	0.340	1.653
Without abnormal fasting glucose level	1.569	10.580	0.001*	4.800
Normal hepatic function	0.166	0.023	0.879	1.181
Normal renal function	4.331	4.161	0.041*	76.050
Normal plasma fibrinogen level	1.970	2.519	0.112	7.167
Normal blood pressure level	3.128	2.733	0.098	22.826

*p values<0.05 are considered significant.

## Discussion

Guillain-Barré syndrome is a rapid-onset weakness and numbness disease caused by the immune system damaging the peripheral nervous system. GBS is usually self-limiting, and most patients either recover completely or only retain minor residual symptoms. But there are still several patients who may face severe outcomes including death. In our study, GBS prognosis is quite favorable; 74.8% got more than 30 points in the MRC score test at discharge. Diabetes, high fasting blood glucose level and high blood pressure at admission, uroschesis, abnormal body temperature, 4requiring ventilator support, disorder of consciousness, no preceding upper respiratory tract infection, low level of blood sodium and albumin, high white blood cell count, high fibrinogen level, and abnormal hepatic and renal function were demonstrated as poor prognostic factors.

There are many subtypes of GBS, such as AIDP, AMAN, MFS, BBE-GBS, and so on, and the proportion of different subtypes varied significantly among different contries and different regions in a same country [18–23]. Our hospital is located in Shanghai which is an east China city, and most our patients are from China, so our study may represent the clinical characteristics and distributions of GBS in China, especially the eastern part of China. In our study, AIDP accounted for 60.4% of all the GBS cases, making it the most common subtype in our hospital. The second one was MFS, accounting for 21.6% of GBS cases, which was similar to 26% in Japan [24] and 25% from the report of Singapore [20]. In sharp contrast, only 7% GBS patients are MFS patients in southwest China [25]. Moreover, AMAN accounted for 8.11% in all GBS patients, CNV accounts for 7.2% and BBE-GBS accounts for only 0.9% of all the patients in our study. Among all the subtypes of GBS, the prognosis of MFS and CNV were the best, and the prognosis of AMAN was worse compared with AIDP in our study, as AMAN is the axonal damage subtype while demyelination is more common among AIDP patients according to electromyogram examinations. González-Suárez et al reported that GBS patients with axon injury were more likely to suffer from respiratory failure [26], leading to poorer prognosis.

The infectious event is described to appear in 40-70% of patients [7, 8, 27–31]. In our series up to 61.8% of cases have had the infectious event, and respiratory infection was the most frequent one among all these infections. We discovered that explicit upper respiratory tract preceding infection was a protective predictor which may help patients get better MRC score at discharge compared with those who had diarrhea or had no explicit preceding infection. This may due to that diarrhea is usually caused by *Campylobacter jejuni (C. jejuni)* and according to a previous study, *C. jejuni* infections exclusively elicit AMAN in East Asia, the axonal damage subtype which has worse prognosis [32], while upper respiratory tract infection is usually caused by other kinds of pathogens.

In our study, we discovered that MFS accounted for 33.33% patients among those who had preceding upper respiratory infection, and only 11.90% patients among those who didn’t have preceding infection. At the same time, CNV accounted for 10.26% patients among those who had preceding upper respiratory infection, and only 7.14% patients among those who didn’t have preceding infection. Since MFS and CNV are the two subtypes that have better prognosis, patients with explicit upper respiratory tract preceding infection showed better prognosis compared with those who without preceding infections.

In our study, patients with diabetes and high fasting blood glucose had poorer prognosis. A previous reports also claimed that diabetes mellitus (DM) is an independent poor prognostic factor for the ability to walk unaided at 3 months after symptom onset [33]. The mechanism is still unclear, but there are several assumptions which may explain. First, some laboratory evidence showed that patients with DM are in a state of chronic low-level inflammation: elevation of various inflammatory markers such as C-reactive protein, tumor necrosis factor and interleukin-6 [34], and this chronic low-level inflammation may lead GBS patients to poor prognosis. Another assumption believed that neurovascular mechanism of DM neuropathy cause bad prognosis for GBS patients, as a chronic state of nerve ischemia in DM may induce partial axonal injury or loss even in subclinical DM neuropathy [35].

GBS has a tendency for dysautonomic features such as bladder dysfunction, abnormal body temperature and hypertension among patients [36, 37]. The underlying mechanisms of urinary dysfunction appear to involve both hypo- and hyperactive lumbosacral nerves caused by GBS [38], and the lumbosacral nerve involvement may relate with poor prognosis. Autonomic dysfunction in patients with GBS reflects dysfunction of sympathic and/or parasympathic innervation, but the exact immunopathological mechanisms remain to be elucidated. Autonomic dysfunction symptoms, such as tachycardia, hypertension, gastrointestinal dysfunction, and bladder dysfunction, can be serious problems as autonomic dysfunction is a predictive poor prognosis factor that may cause sudden death [39–41]. There are two main possible reasons to explain. First, autonomic dysfunction is associated with fatal hypoxia because of respiratory muscle involvement and a long duration of mechanical ventilation and the need for tracheostomy [42, 43]. What’s more, autonomic dysfunction is related to bad prognosis also because of cardiovascular “collapse,” as reported by Clarke et al [44]. However, though related with higher mortality, Samadi M et al found that autonomic dysfunction showed no significant association with motor dysfunction among kids [45], which is different from this report.

According to the previous studies, the incidence of mechanical ventilation in GBS patients is 20~30% according to the previous western reports [46–48]. In our study, this incidence was 14.4%,which is similar to 14.8% of the northeast China report [49], but lower than reports from western countries. The main reason which lead GBS patients to death is respiratory failure [50]. The need of mechanical ventilation and ICU is not only a necessity to those who suffer from respiratory failure, but also a significant sign of respiratory muscle involvement and severe conditions. In accordance with our assumption, our result showed that those who used mechanical ventilation and ICU during hospitalization had worse MRC score at discharge.

Hyponatremia is so common in GBS patient,despite that it is not a classical manifestation of GBS; however, there are series in which are described to be present in 21.5 to 48% of the cases; in our review, it was found in 21.6% of our patients, which is similar to the Northern China study and the British study [51–54]. Hyponatremia is also a predictor of poor prognosis. There may be two possible reasons that make hyponatremia happen: First is the syndrome of inappropriate antidiuretic hormone secretion (SIADH) [53], the possible mechanism could be that SIADH was due to abnormalities of peripheral autonomic afferent fibers arising from vascular stretch receptors or due to increased renal tubular sensitivity to vasopressin. Another reason is cerebral salt wasting syndrome (CSWS) [55], and the possible mechanism involves an inappropriate Brain Natriuretic Peptide secretion upon sympatho-adrenal dysregulation as part of GBS dysautonomia. The treatment principles between SIADH and CSWS are totally different, and the incorrect management of hyponatremia may lead to osmotic demyelinating syndrome. This may explain the poor outcome at discharge of hyponatremia patients compared with those with normal blood sodium level.

Our study also found elevated liver enzyme level indicates poorer prognosis as well. There are two possible reasons. First, liver damage conditions such as infection with hepatitis virus; alcohol abuse; hepatotoxic drugs; recent surgery and so on may influence the systemic health condition significantly, and those who with liver damage may recover more slowly compared with those who have normal hepatic function. What’s more, according to Oomes PG et al’s report, 38% patients showed a plasma alanine aminotransferase elevation, gamma glutamyl transferase elevation, or both or more than 1.5 times the upper limit of normal, of who most were negative for known causes of liver damage [56], and in that study, IVIg treatment seemed to be associated with mild transient liver function disturbances through an unknown mechanism. When admission, some of our patients might have already received IVIg treatment in other hospitals, thus making their liver enzyme level elevated at adimission. Since referral patients’ health conditions are worse than new diagnosed patients generally, the elevated liver enzyme on admission may also be related with worse prognosis in this way.

According to Khajehdehi P et al’s research, acute renal failure can occur commonly in cases with severe GBS patients particularly in those with dysautonomia [57]. What’s more, it has also been reported that patients with GBS can develop acute glomerulonephritis of immune complex origin associated with deterioration of renal function tests. Acute interstitial nephritis is another possibility for renal function deterioration in GBS patients [58]. Elevated Scr and BUN are signs of renal function deterioration and ARF, so that they are related with poorer prognosis in our study.

Our study also found that poor nutrition condition such as low serum albumin level, high coagulation state as high fibrinogen leval, and infection at early stage of GBS such as high white blood cell count, are also predictors of poorer prognosis.

Our work has several limitations. First, as a retrospective study, some clinical parameters which have been reported to be predictors of GBS prognosis were unavailable in our cohort, such as vital capacity and the Peak Flow-test result [59]. What’s more, we didn’t collect the MRC score and other clinical features after patients’ discharge, making the estimation of the long-term prognosis impossible. Some clinical parameters which has been reported as predictors of prognosis in the previous studies but didn’t show statistical differences in our study such as Anti-GQ1b antibody [60] may due to the small sample capacity and the regional/racial differences. Larger and prospective studies will be required.

## Conclusion

AMAN, diabetes, high blood pressure, uroschesis, high body temperature, ventilator support, consciousness disorder, absence of upper respiratory tract preceding infection, hyperglycemia, hyponatremia, hypoalbuminemia, high leukocyte count, hyperfibrinogenemia, abnormal hepatic and renal function were demonstrated as poor prognostic factors. In order to identify patients with bad prognosis at the early stage and get a good better outcome, more attention should be paid to these poor prognostic factors.

## Abbreviations

AIDP: Acute inflammatory demyelinating polyneuropathy
ALT: Alanine aminotransferase
AMAN: Acute motor axonal neuropathy
AST: Aspartate aminotransferase
BBE-GBS: Bickerstaff's brainstem encephalitis overlaps with Guillain-Barre syndrome
BUN: Blood urea nitrogen
CNV: Cranial nerve variant
CSF: Cerebrospinal fluid
CSWS: Cerebral salt wasting syndrome
DM: Diabetes mellitus
GBS: Guillain-Barré syndrome
HFGS: Hughes Functional Grading Scale
ICU: Intensive care unit
IVIg: Intravenous immunoglobulin
MFS: Miller-Fisher syndrome
MRC: Medical Research Council
PE: Plasma exchange
Scr: Serum creatinine
SIADH: Syndrome of inappropriate antidiuretic hormone secretion

## References

[1] van den Berg B, Walgaard C, Drenthen J, Fokke C, Jacobs BC, van Doorn PA (2014). Guillain-Barre syndrome: pathogenesis, diagnosis, treatment and prognosis. Nat Rev Neurol.

[2] Wakerley BR, Yuki N (2013). Infectious and noninfectious triggers in Guillain-Barre syndrome. Expert Rev Clin Immunol.

[3] Hughes RA, Cornblath DR (2005). Guillain-Barre syndrome. Lancet.

[4] Yuki N, Hartung HP (2012). Guillain-Barre syndrome. N Engl J Med.

[5] Yuki N, Hirata K (1998). Preserved tendon reflexes in Campylobacter neuropathy. Ann Neurol.

[6] Kuwabara S, Nakata M, Sung JY, Mori M, Kato N, Hattori T (2002). Hyperreflexia in axonal Guillain-Barre syndrome subsequent to Campylobacter jejuni enteritis. J Neurol Sci.

[7] van Doorn PA, Ruts L, Jacobs BC (2008). Clinical features, pathogenesis, and treatment of Guillain-Barre syndrome. Lancet Neurol.

[8] Vucic S, Kiernan MC, Cornblath DR (2009). Guillain-Barre syndrome: an update. J Clin Neurosci.

[9] Sejvar JJ, Baughman AL, Wise M, Morgan OW (2011). Population incidence of Guillain-Barré syndrome: a systematic review and meta-analysis. Neuroepidemiology.

[10] Hughes RA, Swan AV, Raphaël JC, Annane D, Van KR, van Doorn PA (2007). Immunotherapy for Guillain-Barre syndrome: a systematic review. Brain.

[11] Hughes RA, Swan AV, PAV D (2004). Cochrane Review: Intravenous immunoglobulin for Guillain-Barré syndrome. Cochrane Database of Systematic Reviews.

[12] van Doorn PA (2013). Diagnosis, treatment and prognosis of Guillain-Barre syndrome (GBS). Presse Med.

[13] Dhar R, Stitt L, Hahn AF (2008). The morbidity and outcome of patients with Guillain-Barré syndrome admitted to the intensive care unit. Journal of the Neurological Sciences.

[14] Alshekhlee A, Hussain Z, Sultan B, Katirji B (2008). Guillain-Barre syndrome: incidence and mortality rates in US hospitals. Neurology.

[15] Van DBB, Bunschoten C, van Doorn PA, Jacobs BC. Mortality in Guillain-Barre syndrome. Neurology 2013;80(18):1650. 10.1212/WNL.0b013e3182904fcc 23576619

[16] Van KR, Steyerberg EW, Hughes RA, Swan AV, van Doorn PA, Jacobs BCA (2007). clinical prognostic scoring system for Guillain-Barre syndrome. Lancet Neurology.

[17] Suárez AA, Pessolano FA, Monteiro SG, Ferreyra G, Capria ME, Mesa L (2002). Peak flow and peak cough flow in the evaluation of expiratory muscle weakness and bulbar impairment in patients with neuromuscular disease. Am J Phys Med Rehabil.

[18] Hadden RD, Cornblath DR, Hughes RA, Zielasek J, Hartung HP, Toyka KV (1998). Electrophysiological classification of Guillain-Barre syndrome: clinical associations and outcome. Plasma Exchange/Sandoglobulin Guillain-Barre Syndrome Trial Group. Ann Neurol.

[19] Ma YM, Liu TK, Wong V (2010). Guillain-Barre syndrome in southern Chinese children: 32 year experience in Hong Kong. Pediatrics International Official Journal of the Japan Pediatric Society.

[20] Ng YS, Lo YL, Lim PA (2004). Characteristics and acute rehabilitation of Guillain-Barre syndrome in Singapore. Ann Acad Med Singap.

[21] Doorn PAV, Kuitwaard K, Walgaard C, Koningsveld RV, Ruts L, Jacobs BC. DOI 10.1007/s10875-010-9407-4 IVIG Treatment and Prognosis in Guillain–Barré Syndrome. 2010. 10.1007/s10875-010-9407-4 PMC2883091 20396937

[22] Vucic S (2004). Cairns KDBlack KR, Tick Chong PS, Cros D. Neurophysiologic findings in early acute inflammatory demyelinating polyradiculoneuropathy. Clinical Neurophysiology.

[23] Ho TW, Mishu B, Li CY, Gao CY, Cornblath DR, Griffin JW*, et al.*. Guillain-Barre syndrome in northern China. Relationship to Campylobacter jejuni infection and anti-glycolipid antibodies. Brain 1995;118 ( Pt 3)(3):597. 10.1093/brain/118.3.597 7600081

[24] Mitsui Y, Kusunoki S, Arimura K, Kaji R, Kanda T, Kuwabara S (2015). A multicentre prospective study of Guillain-Barre syndrome in Japan: a focus on the incidence of subtypes. J Neurol Neurosurg Psychiatry.

[25] Zhang G, Li Q, Zhang R, Wei X, Wang J, Subtypes QX (2015). Prognosis of Guillain-Barré Syndrome in Southwest China. Plos One.

[26] Gonzalez-Suarez I, Sanz-Gallego I, Rodriguez DRF, Arpa J (2013). Guillain-Barre syndrome: natural history and prognostic factors: a retrospective review of 106 cases. BMC Neurol.

[27] Telleria-Diaz A, Calzada-Sierra DJ (2002). Guillain Barre syndrome. Rev Neurol.

[28] Aladro-Benito Y, Conde-Sendin MA, Munoz-Fernandez C, Perez-Correa S, Alemany-Rodriguez MJ, Fiuza-Perez MD (2002). Guillain-Barre syndrome in the northern area of Gran Canaria and the island of Lanzarote. Rev Neurol.

[29] A prospective study on the incidence and prognosis of Guillain-Barre syndrome in Emilia-Romagna region, Italy (1992-1993) (1997). Emilia-Romagna Study Group on Clinical and Epidemiological Problems in Neurology. Neurology.

[30] Lyu RK, Tang LM, Cheng SY, Hsu WC, Chen ST (1997). Guillain-Barre syndrome in Taiwan: a clinical study of 167 patients. J Neurol Neurosurg Psychiatry.

[31] Cuadrado JI, de Pedro-Cuesta J, Ara JR, Cemillan CA, Diaz M, Duarte J (2001). Guillain-Barre syndrome in Spain, 1985-1997: epidemiological and public health views. Eur Neurol.

[32] Kuwabara S (2011). Does Campylobacter jejuni infection elicit axonal or demyelinating Guillain-Barre syndrome, or both?. J Neurol Neurosurg Psychiatry.

[33] Bae JS, Kim YJ, Kim JK (2016). Diabetes mellitus exacerbates the clinical and electrophysiological features of Guillain-Barre syndrome. Eur J Neurol.

[34] Hartge MM, Unger T, Kintscher U (2007). The endothelium and vascular inflammation in diabetes. Diab Vasc Dis Res.

[35] Bae JS, Kim OK, Kim JM (2011). Altered nerve excitability in subclinical/early diabetic neuropathy: evidence for early neurovascular process in diabetes mellitus?. Diabetes Res Clin Pract.

[36] Naphade PU, Verma R, Garg RK, Singh M, Malhotra HS, Shankwar SN (2012). Prevalence of bladder dysfunction, urodynamic findings, and their correlation with outcome in Guillain-Barre syndrome. Neurourol Urodyn.

[37] Watson L, Aziz M, Vassallo G, Plant ND, Webb NJ (2014). Bladder dysfunction and hypertension in children with Guillain-Barre syndrome. Pediatr Nephrol.

[38] Sakakibara R, Uchiyama T, Kuwabara S, Mori M, Ito T, Yamamoto T (2009). Prevalence and mechanism of bladder dysfunction in Guillain-Barre Syndrome. Neurourol Urodyn.

[39] Kalita J, Misra UK, Goyal G, Das M (2014). Guillain-Barre syndrome: subtypes and predictors of outcome from India. J Peripher Nerv Syst.

[40] Samukawa M, Hamada Y, Kuwahara M, Takada K, Hirano M, Mitsui Y (2014). Clinical features in Guillain-Barre syndrome with anti-Gal-C antibody. J Neurol Sci.

[41] Zochodne DW (1994). Autonomic involvement in Guillain-Barre syndrome: a review. Muscle Nerve.

[42] Nguyen TN, Badjatia N, Malhotra A, Gibbons FK, Qureshi MM, Greenberg SA (2006). Factors predicting extubation success in patients with Guillain-Barre syndrome. Neurocrit Care.

[43] Lawn ND, Wijdicks EF (1999). Tracheostomy in Guillain-Barre syndrome. Muscle Nerve.

[44] CLARKE E, BAYLISS RI, COOPER R (1954). Landry-Guillain-Barre syndrome: cardiovascular complications; treatment with A.C.T.H. and cortisone. Br Med J.

[45] Samadi M, Kazemi B, Golzari OS, Barzegar M (2013). Assessment of autonomic dysfunction in childhood guillain-barre syndrome. J Cardiovasc Thorac Res.

[46] Hahn AF (2001). The challenge of respiratory dysfunction in Guillain-Barre syndrome. Arch Neurol.

[47] Lawn ND, Fletcher DD, Henderson RD, Wolter TD, Wijdicks EF (2001). Anticipating mechanical ventilation in Guillain-Barre syndrome. Arch Neurol.

[48] Sharshar T, Chevret S, Bourdain F, Raphael JC (2003). Early predictors of mechanical ventilation in Guillain-Barre syndrome. Crit Care Med.

[49] Wu X, Li C, Zhang B, Shen D, Li T (2015). Liu K*, et al.*. Predictors for mechanical ventilation and short-term prognosis in patients with Guillain-Barre syndrome. Crit Care.

[50] Durand MC, Porcher R, Orlikowski D, Aboab J, Devaux C, Clair B (2006). Clinical and electrophysiological predictors of respiratory failure in Guillain-Barre syndrome: a prospective study. Lancet Neurol.

[51] Wang Y, Liu J (2015). Hyponatremia is a predictor for poor outcome in Guillain-Barre syndrome. Neurol Res.

[52] Colls BM (2003). Guillain-Barre syndrome and hyponatraemia. Intern Med J.

[53] Saifudheen K, Jose J, Gafoor VA, Musthafa M (2011). Guillain-Barre syndrome and SIADH. Neurology.

[54] Ng KK, Howard RS, Fish DR, Hirsch NP, Wiles CM, Murray NM (1995). Management and outcome of severe Guillain-Barre syndrome. QJM.

[55] Lenhard T, Grimm C, Ringleb PA (2011). Renal salt wasting as part of dysautonomia in Guillain-Barre syndrome. J Neurol Neurosurg Psychiatry.

[56] Oomes PG, van der Meche FG, Kleyweg RP (1996). Liver function disturbances in Guillain-Barre syndrome: a prospective longitudinal study in 100 patients. Dutch Guillain-Barre Study Group. Neurology.

[57] Khajehdehi P, Shariat A, Nikseresht A (1998). Acute renal failure due to severe Landry-Guillain-Barre syndrome. Nephrol Dial Transplant.

[58] Buysen JG, Houthoff HJ, Krediet RT, Arisz L (1990). Acute interstitial nephritis: a clinical and morphological study in 27 patients. Nephrol Dial Transplant.

[59] Suarez AA, Pessolano FA, Monteiro SG, Ferreyra G, Capria ME, Mesa L (2002). Peak flow and peak cough flow in the evaluation of expiratory muscle weakness and bulbar impairment in patients with neuromuscular disease. Am J Phys Med Rehabil.

[60] Kaida K, Kusunoki S, Kanzaki M, Kamakura K, Motoyoshi K, Kanazawa I (2004). Anti-GQ1b antibody as a factor predictive of mechanical ventilation in Guillain-Barre syndrome. Neurology.

